# Childhood Anemia in a Malaria-Endemic Region: The Haptoglobin Genotype Connection

**DOI:** 10.1371/journal.pmed.0030227

**Published:** 2006-05-02

The World Health Organization estimates that malaria kills an African child every 30 seconds. Many of these children die because they develop severe anemia (a deficiency of red blood cells). As many as 5 million cases of severe malarial anemia occur in African children every year, and 13% of these cases are fatal. Turning the statistics around, more than half of young children in African countries where malaria is endemic (constantly present) are anemic. Nutritional deficiencies and various infections account for some of this disease burden, but malaria is one of the most important factors contributing to anemia.

The malaria parasite destroys red blood cells (a process called hemolysis) as part of its life cycle, releasing hemoglobin (Hb)—an iron-containing protein that carries oxygen around the body—into the circulation. Free Hb can cause oxidant stress, which is itself associated with anemia in malaria. An important modulator of such stress is a serum protein called haptoglobin (Hp), which captures Hb during hemolysis.

Hp exists in three molecular forms that are genetically determined by two variants (alleles) of a single gene. People who have two copies of the *Hp ^1^* allele make only Hp1-1, a homodimeric protein. People with two copies of the *Hp ^2^* allele (the *Hp ^2/2^* genotype) make Hp2-2, a large circular polymer, and those with one copy of each allele make the linear polymer Hp1-2 in addition to these two forms. The functional properties of the three Hp forms are somewhat different. In particular, Hp2-2 binds Hb much less tightly than the other forms. Sarah Atkinson and her colleagues reasoned, therefore, that the *Hp ^2/2^* genotype might be a risk factor for anemia in children in malaria-endemic areas. To test their hypothesis, they measured Hb levels in Gambian children at the start and end of the malaria season, and now report in a new study that, as predicted, the *Hp ^2/2^* genotype is associated with seasonal childhood anemia in this population.

Most cases of malaria in The Gambia occur between September and December, so the researchers recruited 780 children aged two to six years from ten Gambian villages in July 2001, determined their *Hp* genotypes, assessed their blood Hb and serum Hp concentrations and iron status, and determined whether they were infected with malaria parasites. These variables were re-measured at the end of the malaria season. In addition, the researchers determined two other genetic polymorphisms that might influence Hb levels over the malaria season—an *Hb* variant that causes sickle cell anemia *(HbS)*, and *glucose-6 phosphate dehydrogenase (G6PD)* gene variants associated with hemolytic anemia.

Atkinson and her colleagues first analyzed their study population in terms of their *Hp* genotype. This univariate (single) analysis included 671 children—a few children were not included because of incomplete data. Baseline hb levels were not affected by *Hp* genotype, but the average drop in hb was 8.9 g/l in the 17% of children with the *Hp ^2/2^* genotype compared with only 5.1 g/l in children with the other genotypes. By contrast, the magnitude of the drop in Hb levels over the season was not affected by *HbS* or *G6PD* genotype—two other genetic traits that affect the red blood cells. Because multiple factors influence Hb concentrations (for example, recent infection with malarial parasites and iron status), the researchers also did a multiple regression analysis of their data to test the effect of all such factors on Hb levels at the end of the malaria season. There were 565 children who had data complete enough for this more detailed analysis, and, once again, the *Hp* genotype emerged as a risk factor for anemia, even after adjusting for other factors that affect Hb levels.

Atkinson and her colleagues suggest that the association between *Hp* genotype and seasonal childhood anemia may reflect the reduced ability of the Hp2-2 polymer to scavenge free Hb and its bound iron after malaria-induced hemolysis. They also discuss why *Hp ^2^*, a potentially detrimental allele, should be common in The Gambia, where malaria is endemic. *Hp ^2^* arose from *Hp ^1^* about 2 million years ago, and its subsequent spread across the world seems to have been driven by a strong genetic pressure, such as exposure to a life-threatening disease. The authors suggest that malaria may be one of the diseases that helped to select for the *Hp ^2^* allele; it is possible that the *Hp ^2^* allele may provide protection from life-threatening malaria, albeit at the expense of impaired hematological recovery from mild and asymptomatic malaria. In a related Perspective (DOI: 10.1371/journal.pmed.0030200), Stephen Rogerson expands on the possible mechanisms of Hp-related anemia, and considers what the wider health implications of this study might be.

## 

**Figure:**
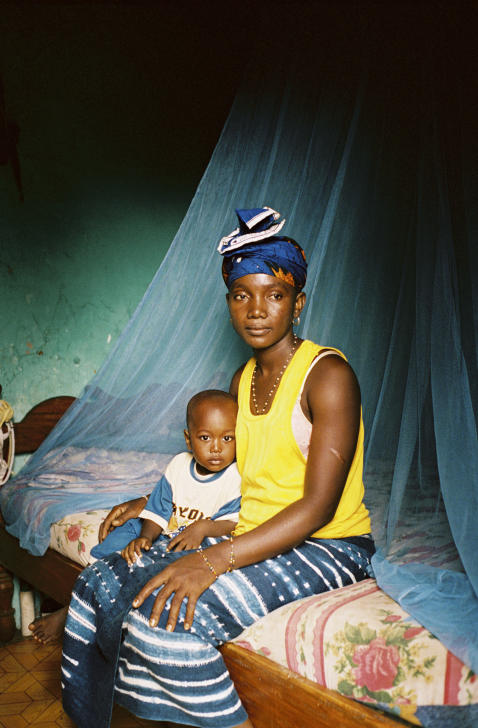
Bed nets are essential in malaria-endemic regions such as The Gambia

